# Geographic and socioeconomic inequalities in the coverage of contraception in Uttar Pradesh, India

**DOI:** 10.1186/s12978-024-01784-3

**Published:** 2024-04-11

**Authors:** Shiva S Halli, Mohd Tauheed Alam, Vasanthakumar Namasivayam, Ravi Prakash, Preeti Anand, James Blanchard, Fernando Wehrmeister

**Affiliations:** 1 Department of Community Health Sciences, Institute for Global Public Health,, University of Manitoba, Winnipeg, Canada; 2 India Health Action Trust, Lucknow, Uttar Pradesh India

**Keywords:** Contraception, Coverage, Inequalities, Administrative Divisions, Uttar Pradesh, Demand Met, गर्भनिरोधक, व्यापकता, असमानताएं, मण्डल, उत्तर प्रदेश, संतुष्ट मांग

## Abstract

**Background:**

Uttar Pradesh (UP) is the most populous state in India, with a historically lower level of family planning coverage than the national average. In recent decades, family planning coverage in UP has significantly increased, yet there are considerable geographic and socio-economic inequalities.

**Methods:**

The data used for the study is derived from a cross-sectional quantitative survey of 12,200 currently married women conducted during December 2020–February 2021 in UP by the Technical Support Unit. Univariate and bivariate analyses were performed and equiplots were used to make visualizing inequalities easy.

**Results:**

The findings of the study reveal significant variation in family planning coverage indicators amongst currently married women in reproductive ages by administrative divisions in UP. For instance, in the Jhansi division, it was 72.4%, while in Faizabad, it was 39.3%. Jhansi division experienced the highest modern contraceptive coverage with the lowest inequity compared to other divisions. However, the range of coverage within the division by Accredited Social Health Activist (ASHA) areas is 25% to 75%. In fact, for some ASHA areas in the Jhansi division, the family planning demand satisfied for modern contraception ranged from more than 85% to less than 22%. On the other hand, the Gonda division with the lowest coverage and lowest inequity for demand satisfied for modern contraception has some ASHA areas with less than 5% and some with more than 36%. The study also revealed intersectionality of education, wealth, place of residence and geographic divisions in identifying inequity patterns. For instance, in case of Mirzapur and Varanasi, the demand satisfied among the illiterates was 69% and the corresponding percentage for literates was 49%. With respect to place of residence, Basti division, where the coverage for modern contraception is extremely low, demand satisfied for modern contraceptive methods is 16.3% among rural residents compared to 57.9% in the case of urban residents.

**Conclusions:**

The findings showed inequality in the modern family planning methods coverage in UP in both best and worst performing divisions. The inequalities exist even in extremely small geographies such as ASHA areas. Within the geographies as well, the socio-economic inequalities persisted. These inequalities at multiple levels are important to consider for effective resource allocation and utilization.

## अमूर्त

**पृष्ठभूमि:**

उत्तर प्रदेश (यूपी) भारत का सबसे अधिक आबादी वाला राज्य है, जहां परिवार नियोजन व्यापकता का स्तर ऐतिहासिक रूप से राष्ट्रीय औसत से कम है। हालाँकि हाल के दशकों में यूपी में परिवार नियोजन व्यापकता में उल्लेखनीय वृद्धि हुई है, फिर भी इसमें काफी भौगोलिक और सामाजिक-आर्थिक असमानताएँ हैं।

**विधि:**

अध्ययन के लिए उपयोग किया गया डेटा यूपी तकनीकी सहयोग इकाई द्वारा आयोजित 12,200 वर्तमान में विवाहित महिलाओं के क्रॉस-सेक्शनल मात्रात्मक सर्वेक्षण से लिया गया है, और STATA का उपयोग करके विश्लेषण किया गया है | असमानताओं को दर्शाने के लिए equiplots का उपयोग किया गया है |

**परिणाम:**

अध्ययन के निष्कर्षों से यूपी के मंडलों मैं प्रजनन आयु में वर्तमान में विवाहित महिलाओं के बीच परिवार नियोजन व्यापकता संकेतकों में महत्वपूर्ण भिन्नता है। उदाहरण के लिए, झाँसी मंडल में यह 72.4% था जबकि फ़ैज़ाबाद में यह 39.3% था। उदाहरण के लिए झाँसी मण्डल में अन्य मंडलों की तुलना में सबसे कम असमानता के साथ उच्चतम आधुनिक गर्भनिरोधक व्यापकता है | हालाँकि, आशा क्षेत्रों द्वारा प्रभाग के भीतर व्यापकता की सीमा 25% से 75% है। वास्तव में, झाँसी मण्डल के कुछ आशा क्षेत्रों में आधुनिक गर्भनिरोधक के लिए परिवार नियोजन की संतुष्ट मांग 85% से अधिक से लेकर 22% से कम थी। दूसरी ओर, आधुनिक गर्भनिरोधक के लिए संतुष्ट मांग के लिए सबसे कम कवरेज और सबसे कम असमानता वाले गोंडा मण्डल में कुछ आशा क्षेत्र हैं जहां संतुष्ट मांग 5% से कम और 36% से अधिक है। अध्ययन में असमानता पैटर्न की पहचान करने में शिक्षा, संपत्ति, निवास स्थान और भौगोलिक विभाजन की अंतर्संबंधता का भी पता चला। उदाहरण के लिए, मिर्ज़ापुर और वाराणसी के मामले में, निरक्षरों के बीच संतुष्ट मांग 69% है और साक्षरों के लिए यह प्रतिशत 49% है। निवास स्थान के संबंध में, बस्ती मंडल जहां आधुनिक गर्भनिरोधक के लिए कवरेज बेहद कम है, ग्रामीण निवासियों के बीच आधुनिक गर्भनिरोधक तरीकों की संतुष्ट मांग 16.3% है, जबकि शहरी निवासियों के मामले में यह 57.9% है।

**निष्कर्ष:**

वर्तमान अध्ययन का मुख्य निष्कर्ष यह है कि उत्तर प्रदेश में सबसे अच्छे और सबसे खराब प्रदर्शन करने वाले मंडलों में भी आधुनिक परिवार नियोजन विधियों के कवरेज में असमानता मौजूद है। आशा क्षेत्रों जैसे अत्यंत छोटे भौगोलिक क्षेत्रों में भी असमानता मौजूद है। भौगोलिक क्षेत्रों में सामाजिक-आर्थिक असमानताएँ भी हैं। प्रभावी संसाधन आवंटन और उपयोग के लिए नीति निर्माताओं के लिए विचार करने के लिए ये महत्वपूर्ण कारक हैं।

## Background

Uttar Pradesh (UP) is the most populous state in India with an estimated population of 235 million, accounting for 17% of India’s population, with a district- wide heterogeneity in socio-economic status [1, 2]. It is also one of the most socio-economically disadvantaged states; over three-fourths (76%) of the population is rural. The State is divided into 18 administrative Divisions, 75 Districts, 820 Blocks, and around 105,000 villages. To control its growing population, until 1990, there was no clear differentiation in policies between UP and India. However, the Government of UP, jointly with the Government of India with assistance from the United States Agency for International Development (USAID), established an autonomous body in 1992, the State Innovations in Family Planning Services Project Agency (SIFPSA), to review and revise the state’s population policy [3]. SIFPSA developed many innovations such as community-based distributors for contraceptives and clinical outreach camps in low resource settings. In 2000, the Government of India formulated the National Population Policy to achieve population stabilization by 2045 by addressing unmet need for family planning. A similar exercise was carried out in UP with innovative family planning strategies by SIFPSA. For instance, an environment of informed advocacy was created by providing evidence on expanding method choice and the impact of improved family planning services. When Government of India introduced National Rural Health Mission (NRHM) in 2005 to improve the health systems in the states, many of SIFPSA’s interventions were absorbed into NRHM programmes [4], including decentralized planning. The Family Planning Program in UP has received a big boost since 2013 by providing improved family planning services through the newly established Technical Support Unit by the University of Manitoba (UoM) in collaboration with the India Health Action Trust (IHAT).

Following an evidence-based approach, IHAT enabled women to use contraceptive information, services, and supplies; in hard-to-reach areas, regular family planning camps (fixed day services) were organized especially to meet the growing demand for terminal methods. Despite the government’s efforts, the five rounds of the National Family Health Survey (NFHS) have revealed that the unmet need for family planning is higher in UP in comparison to the national average of unmet need. This was 30.1% in UP compared to 19.5% in India in 1992-93 [5]. The difference in unmet need between India and UP has been reduced in the recent years to 12.9% in UP compared to 9.4% in India in 2019-21 [6]. Although unmet need is most used to understand how differential demand for fertility reduction is achieved, the “true coverage indicator” is the demand met for modern family planning methods [7]. The increase in the proportion of women whose demand for contraception has been met by traditional method of contraception has increased much more rapidly in UP compared to India during period of 1992-3 to 2019-21. For instance, the difference between UP and India in 1992-93 in the case of demand met by any method was as high as more than 30 percentage points but it reduced to single digit of 5 percentage points in 2019-21 [5, 6]. However, the increase in demand met by modern contraceptive methods has not increased at the same pace in UP. To be specific, the difference in demand met by modern methods between UP and India was 25 percentage points in 1992-93 and only declined to 15 in 2019-2021 (NFHS-5).

The 2030 Agenda for Sustainable Development Goals - “Leave No One Behind (LNOB)” also includes reproductive and child health [8]. A state like Uttar Pradesh needs to recognize the profound transformation required to address the emerging challenges of LNOB. The challenges include widespread regional disparities and urban-rural gaps in accessing and utilizing of reproductive health services including modern contraceptive methods. There are also inequalities in the coverage of modern family planning methods due to socio-economic differences among women sub-groups. The evidence in India suggests that higher education of women is associated with modern spacing methods, and among women with no formal education or engaged in agricultural activities, the popular method of contraception is sterilization [9]. It is possible that women with more education may have better access to and sufficient knowledge about the efficacy of modern spacing methods in controlling fertility. Previous research also found that higher education increases the odds of adopting a traditional method instead of sterilization [10]. On the other hand, women with little or no education and from poor economic conditions are likely to access freely available modern contraceptives in public sectors [11]. The reason poor women end up with sterilization is because the services offered through public sectors primarily focus on promoting permanent methods [12]. The higher prevalence of sterilization was also found among women from Scheduled Caste and Scheduled Tribe [13]. Moreover, the research also has shown that inequalities in the provision of family planning information, particularly for women from socio-economically backward communities, are disadvantaged in terms of better care and information [14]. It is important to note that the data from the National Family Health Surveys have shown more than 30% of the sterilized women reported that they were not informed that it is a non-reversible method, and nearly 70% of women reported that they were not informed about the side effects of the method [13]. Moreover, those women who adopted sterilization at a very young age, especially those without a son and those who had experienced child loss, regretted their consent to sterilization [15].

It also entails new ways of implementing programmes and monitoring and evaluating interventions needed for spacing and limiting children to improve mother and child health. This means moving beyond assessing average and aggregate progress and ensuring that all population groups experience access and utilize reproductive health services. For this, there is a need for a suitable framework that focuses on measuring family planning equity. While some existing frameworks describe the factors affecting health equity [16, 17], there is a need for a framework to identify the program layer, which contributes to the maximum equity drop so that the smaller regions can be identified and improvement measures undertaken despite the underlying social determinants of reproductive health. One such framework was recently developed based on the experience of maternal and child health programs in UP [18]. With the help of this framework, it is hoped to identify modern contraceptive coverage gaps between and within administrative divisions in UP and determine where the largest gaps exist to help prioritize and plan the most relevant interventions by planners and policymakers to reduce unmet demand for modern family planning methods in Uttar Pradesh.

## Data and methods

### Data source

The data used for the study is derived from a cross-sectional quantitative survey of 12,200 currently married women (CMW) in reproductive ages, 15-49, conducted in Uttar Pradesh by the UP Technical Support Unit (UP TSU) during December 2020-February 2021 [16]. It was a random sample to represent UP’s 18 divisions, 75 districts, 820 blocks and more than 97 thousand villages. The National Family Health Survey -5 (NFHS-5) conducted by International Institute for Population Sciences (IIPS) between 2019-2021 were also used for comparison [6]. NFHS-5 is a nationally representative cross-sectional survey including a representative sample of households throughout India. The survey also provides state and district-level estimates of demographic, health, socioeconomic status, and program dimensions, which are critical for implementing the desired changes in demographic and health parameters. Stratified, two-stage sampling is primarily used intheNFHS-5 survey to obtain a representative sample of households. Probability proportional to size (PPS) was used to select the households from all states and Union Territories. Within each rural stratum, villages were selected from the sampling frame based on the 2011 Census with PPS. In urban areas, the Census Enumeration block (CEB) was selected based on data from the census of India. In the second phase, households were selected using a Systematic Random Sampling design from selected primary sampling units in rural and urban clusters. From the selected households, the respondents were selected. The survey represents all the districts of all the States and Union Territories of India. The household sample size in the survey consists of 636,699 households. A carefully selected well trained interviewers collected information from the women respondents using a computer-assisted personal interviewing (CAPI) electronic device. Detailed information on the survey methodology, the design and the sampling plan are provided in the NFHS-5 report [6]. For this analysis, data of Uttar Pradesh State covering 62,675 currently married women was used. 

#### Sample size and sampling design of UP family planning survey

The sample size for this study was determined using the divisional-level modern contraceptive prevalence rate (mCPR) of NFHS-4 (2015–16). The sample size within each division was decided based on proportional representations of the 2011 Census of India in rural and urban areas. Accredited Social Health Activist (ASHA), a female health activist who caters health-related services to about 1000 population in 250-300 households, was considered as a Primary Sampling Unit (PSU). In total 508 Primary Sampling units (394 rural and 114 urban) were selected across the state. Within the selected division, a two-stage sampling technique was used in rural and urban areas. In the first stage, based on the number required of PSUs was selected using a list of Census villages and Census Enumeration Blocks (CEBs) in urban areas. In the second stage, using a Systematic Random Sampling method, approximately 27 households in each PSU were selected from a household sampling frame prepared before the survey. Written consent was obtained from all the selected adult currently married women aged 15-49 (CMWs) from the households before collecting the information. In the case of the CMWs aged 15-17 years, written consent was obtained from their husbands/heads of the households. A detailed description of the methodology can be found in a published research paper in BMC Reproductive Health [2].

### Measures

The main outcome measure was family planning demand satisfied by modern methods (female sterilization, male sterilization, IUCD- Copper-T/ Loop, Injectables/ *Antra*, Pills, *Chhaya*/ Centchroman, emergency contraception, male condom, female condom, standard days method, lactational amenorrhea method) as compared to other traditional methods (rhythm / withdrawal / other traditional methods such as periodic abstinence). The rationale for using the demand satisfied instead of usual indicator family planning unmet need is that the unmet need measure does not capture behaviour or intent because the computation of unmet need includes women in reproductive ages irrespective of whether they are sexually active or not and using the modern methods or not. [7]. In the study, it was argued that women can consciously decide not to use contraception as they may not be consistently sexually active or may not like it due to side effects. The demand for family planning satisfied with modern contraceptive methods is an improved measure of demand met with modern contraceptive methods (mCPR/mCPR+ Unmet need) as it reflects voluntarism and informed choice. The indicator does not set contraceptive prevalence or fertility targets but emphasizes the imperative to satisfy individuals' and couples' preferences about the number and timing of children. However, the above computational formula was modified by including the prevalence of traditional methods since the use of traditional methods is much higher in UP and, a considerable proportion of women use this as a means of spacing or limiting their family size, hence, the revised formula is (mCPR/(mCPR+ Unmet need+ Prevalence of Traditional methods)). An additional table was provided in the appendix using the data from the most recent Family Planning (FP) Survey in UP to provide an idea about the demand satisfied and unmet need for UP.

In addition to FP demand satisfied by modern contraceptive methods, other coverage indicators considered are FP total unmet need, FP unmet need for limiting (as these are the pregnant/postpartum amenorrheic women who did not want current pregnancy/last child or non-pregnant women who wants no more children in future), FP unmet need for spacing (pregnant/postpartum amenorrheic women who want current pregnancy/last child later or non-pregnant women who wanted next child after 2 years or unsure about the next child or its’ timing), mCPR and CPR. Women’s education, place of residence and wealth quantiles are used to measure socio-economic inequities by administrative divisions [19]. To measure socio-economic inequalities, the indicators were FP demand satisfied by modern methods, modern contraceptive users, and traditional FP methods users (rhythm method, withdrawal, periodic abstinence). Since, there has been significant increase in usage of traditional family planning methods in UP, the two coverage indicators, one combining traditional and modern family planning methods and other representing only modern methods, are Contraceptive Prevalence Rate (CPR) and Modern Contraceptive Prevalence Rate (mCPR) respectively also considered.

The trained female research investigators collected detailed information in the local language on household socio-economic conditions along with details of family members. On the selected currently married women, the individual woman’s questionnaire was administered to collect information on the demographic characteristics, reproduction, marriage and cohabitation, contraception, and fertility history.

### Analysis

Considering the size of the population of UP and the small sample size of 12,200, to have an idea about its representativeness, the sample background characteristics were compared with the large sample of the National Family Health Survey-5, since both the surveys were conducted almost at the same time. To incorporate smaller geographies such as ASHA areas (PSUs) with coverage and family planning inequity, unweighted mean average difference from the mean were calculated considering all PSUs. It was then plotted as a quadrant plot and the four groups created were: Low Coverage – Low Inequality (LC-LI), Low Coverage – High Inequality (LC-HI), High Coverage – High Inequality (HC-HI) and High Coverage- Low Inequality (HC-LI). To define high or low levels of coverage or inequality, we calculated the median, considering all divisions, of both measures. Above the median was considered high and below the median was considered low. To examine socio-economic inequalities within divisions, education and wealth quantile variables were re-groped into two categories (because of small sample) based on table 1. The two categories of education were no education and some education (includes all other categories of education except illiterates in table 1). Similarly, two categories of a wealth quantile variable were poorest (poor) and non-poor (rich-includes remaining four categories of a wealth quintile variable from Table 1).

Data analysis was conducted using Statistical Software (STATA) 15.0 and applied sampling weights where applicable. To examine inequity in family planning coverage between and within administrative divisions as well as by socio-economic characteristics in UP, equiplots were used as equiplots are tools to easily visualize inequality. Equiploter creator tool, based on the language R, was used to draw plots. In equiplots, each dot represents the coverage in a specific subgroup and the gap between dots indicates the inequality in the coverage.

## Results

Table 1 provides the comparable profile of the currently married women surveyed in UP in the most recent Family Planning (FP) Survey and the National Family Health Survey-5. Both the surveys provided very similar profile in terms of background characteristics such as age, parity, education, caste, religion, place of residence and wealth quantile. More specifically, according to FP Survey in UP, the percentage of currently married women in the age group 15-24 was little more than the NFHS-5 data set, 21% and 17% respectively. (This was mainly because sample size was boosted for younger women to have sufficient sample and make reliable estimates for the young women). Similarly, the sampled women in the FP Survey percentage are more in the 4+ parity category compared to the corresponding NFHS-5 sampled women 4+ parity category, 32% and 24% respectively. In rest of the age groups, parity, education of both husband and wife, caste, religion, and place of residence percentages of both the surveys are similar except for higher representation of husbands of the respondents in ten years or more education category in the FP survey.

**Table 1:** Percentage distribution of currently married women by background characteristics, Uttar Pradesh, India

**Age Group**	**UPTSU FP Survey**	**NFHS-5**
15-24	21	17
25-29	21	21
30-34	18	18
35-39	16	17
40-49	26	27
**Parity**		
0	10	10
1	15	16
2	23	27
3	21	23
4+	32	24
**Woman education**		
Illiterate	41	38
1-9th Grades	29	30
10th standard or higher	30	32
**Husband education**		
Illiterate	19	27
1-9th Grades	34	40
10th standard or higher	47	33
**Caste**		
SC/ST	29	26
OBC	54	54
Others	17	20
**Religion**		
Muslim	15	16
Hindu	85	84
**Place of residence**		
Urban	23	24
Rural	77	76
**Wealth quintile**		
Poorest	17	22
Poor	19	24
Middle	21	19
Rich	22	17
Richest	21	18
Total Sample	12,200	62,675

**Fig. 1:**
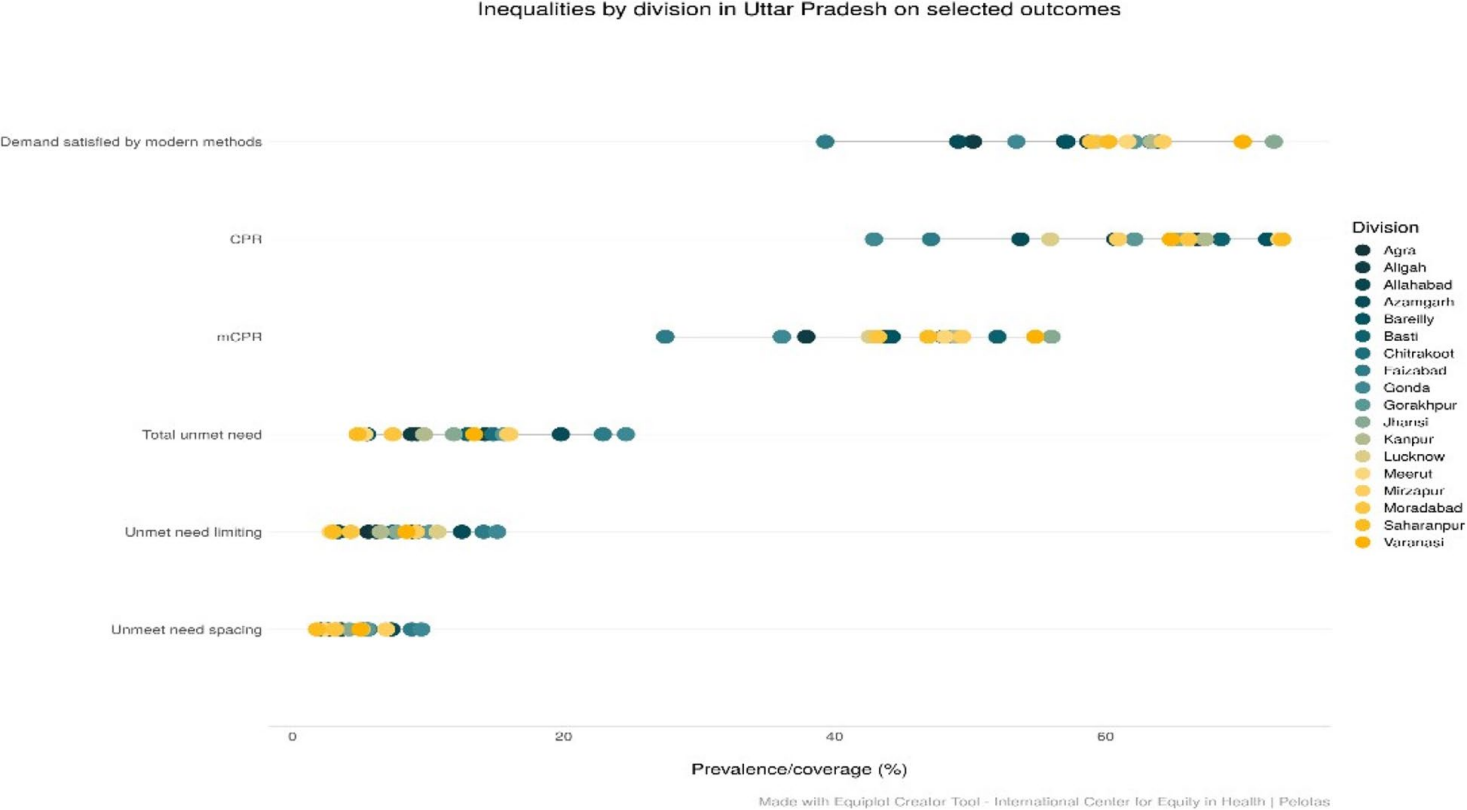
Divisional level inequalities in family planning coverage indicators in Uttar Pradesh, India, 2021

Figure 1 reveals significant variation in family planning coverage indicators amongst currently married women in reproductive ages by administrative divisions in UP. Irrespective of coverage indicators, the pattern is the same. From the figure, the demand satisfied varies significantly between the divisions: In Jhansi division, it was 72.4%, while in Faizabad, it was 39.3% (Actual percentage are available on request). The range for unmet needs also varies from 4.8% in Saharanpur division to 24.6% in Gonda division. Similar patterns can be seen from the figure if the unmet need is observed separately for limiting and spacing of children. The two indicators, one combining the traditional and modern contraceptive methods (CPR) and other representing the modern methods (mCPR) shown significant variation in family planning coverage by divisions, Gonda and Ayodhya divisions are the most disadvantaged divisions irrespective of CPR and mCPR.

**Fig. 2:**
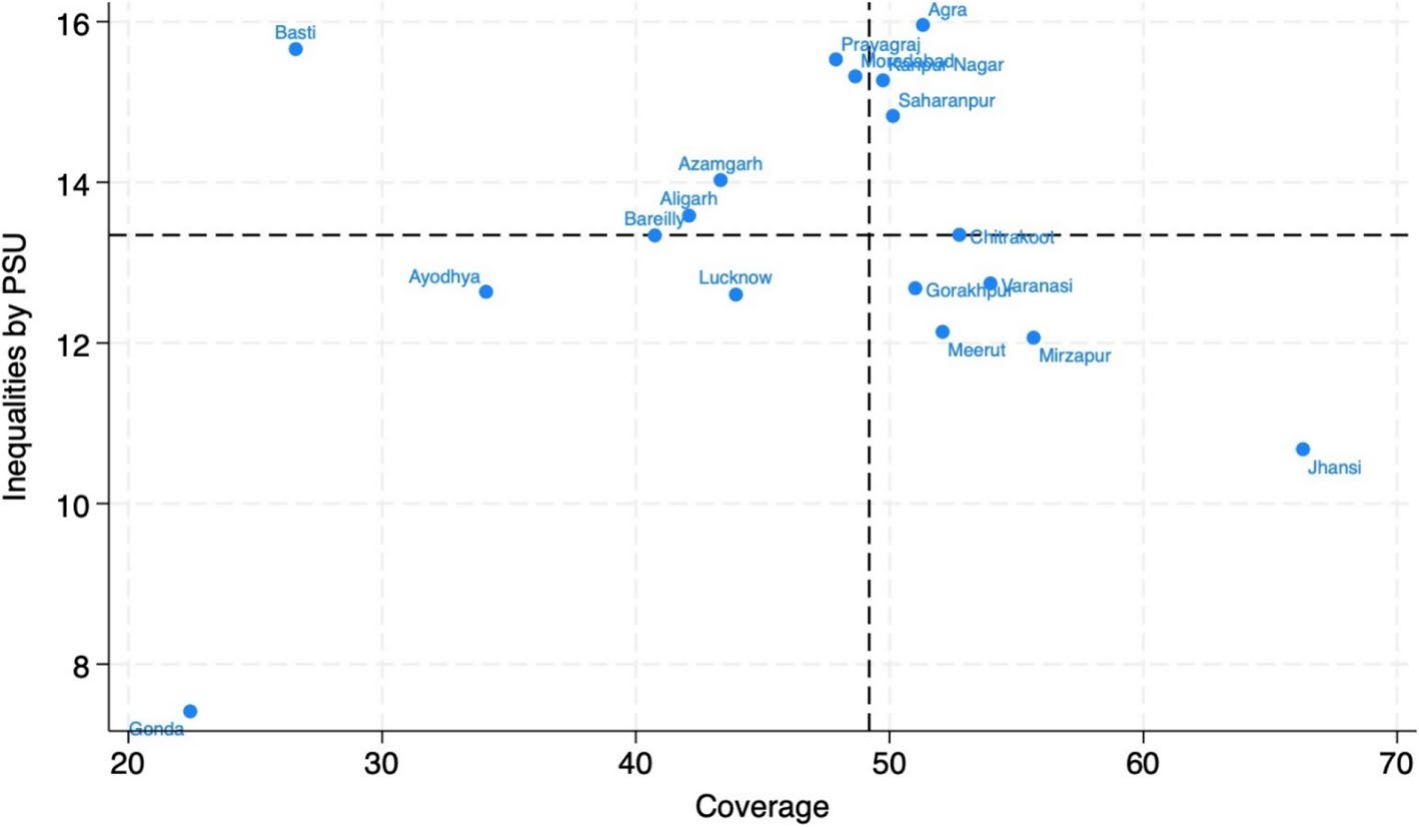
Distribution of divisional inequalities in demand satisfied by modern family planning methods in Uttar Pradesh, India, 2021 (The lines in the Y and X axes represent the median in the coverage (x axis) and the median values in the inequality measure (y axis))

Figure 2 presents distribution of divisional inequalities in demand satisfied by modern family planning methods into four quadrants using median as a cutoff point– Low coverage – Low Inequality (LC-LI), Low Coverage – High Inequality (LC-HI), High Coverage – High Inequality (HC-HI) and High Coverage- Low Inequality (HC-LI). Though there are five divisions in the quadrant with HC-LI, Jhansi division is with highest family planning demand satisfied with lowest inequality. Similarly, in the quadrant LC-HI there are five divisions, but Basti is with the lowest family planning coverage with one of the highest inequalities. In Gonda division both the demand satisfied by modern contraception and corresponding inequity are extremely low (LC-LI). It is also observed that the coverage of demand satisfied by modern contraception in Prayag Raj, Moradabad, Kanpur Nagar and Saharanpur divisions is around median level, but inequality is much above the median. Agra’s divisional performance is with slightly above the median level of family planning demand satisfied with highest inequality.

**Fig. 3:**
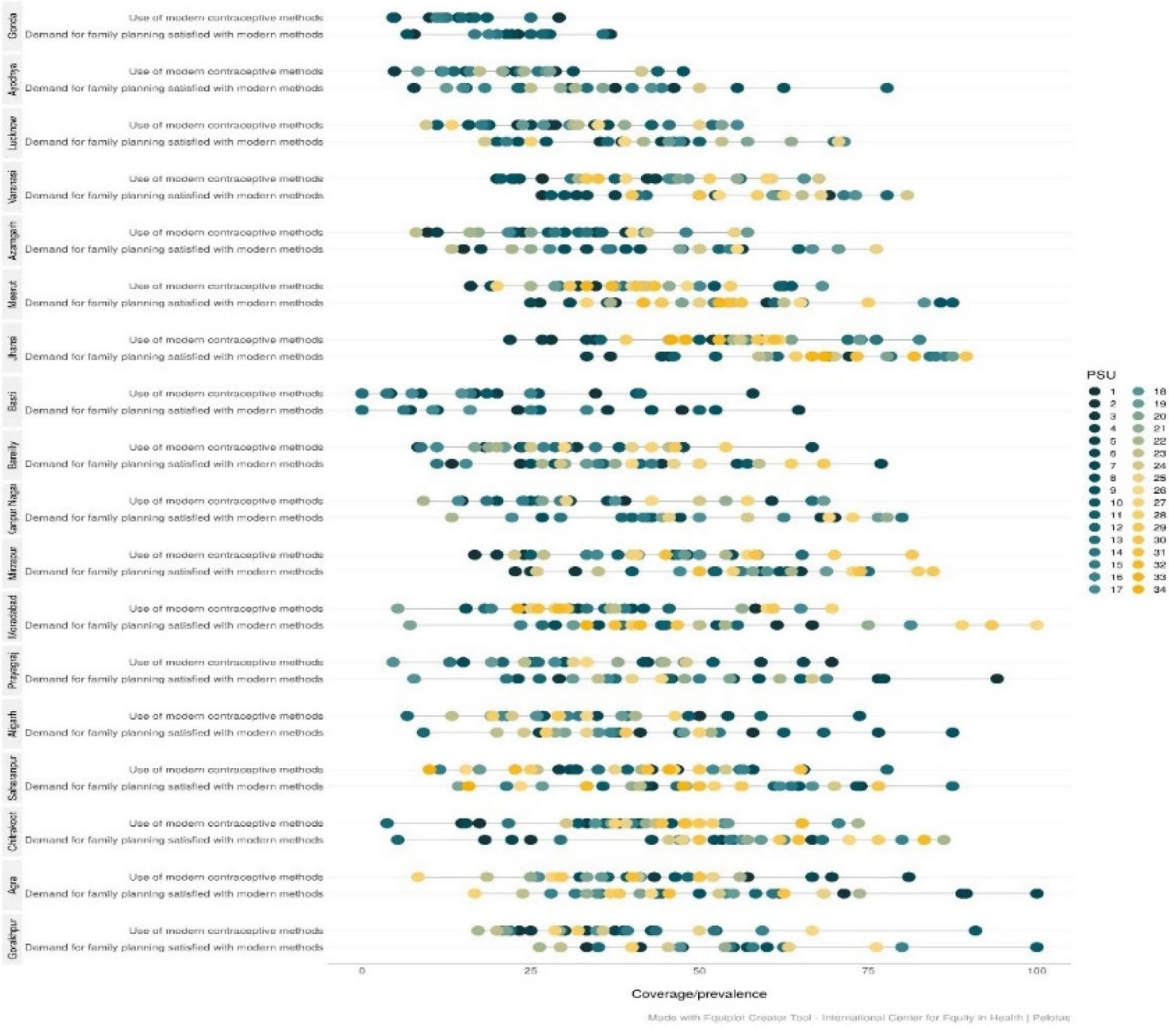
Distribution of within division inequalities in demand satisfied by modern contraception in Uttar Pradesh, India, 2021

**Fig. 4:**
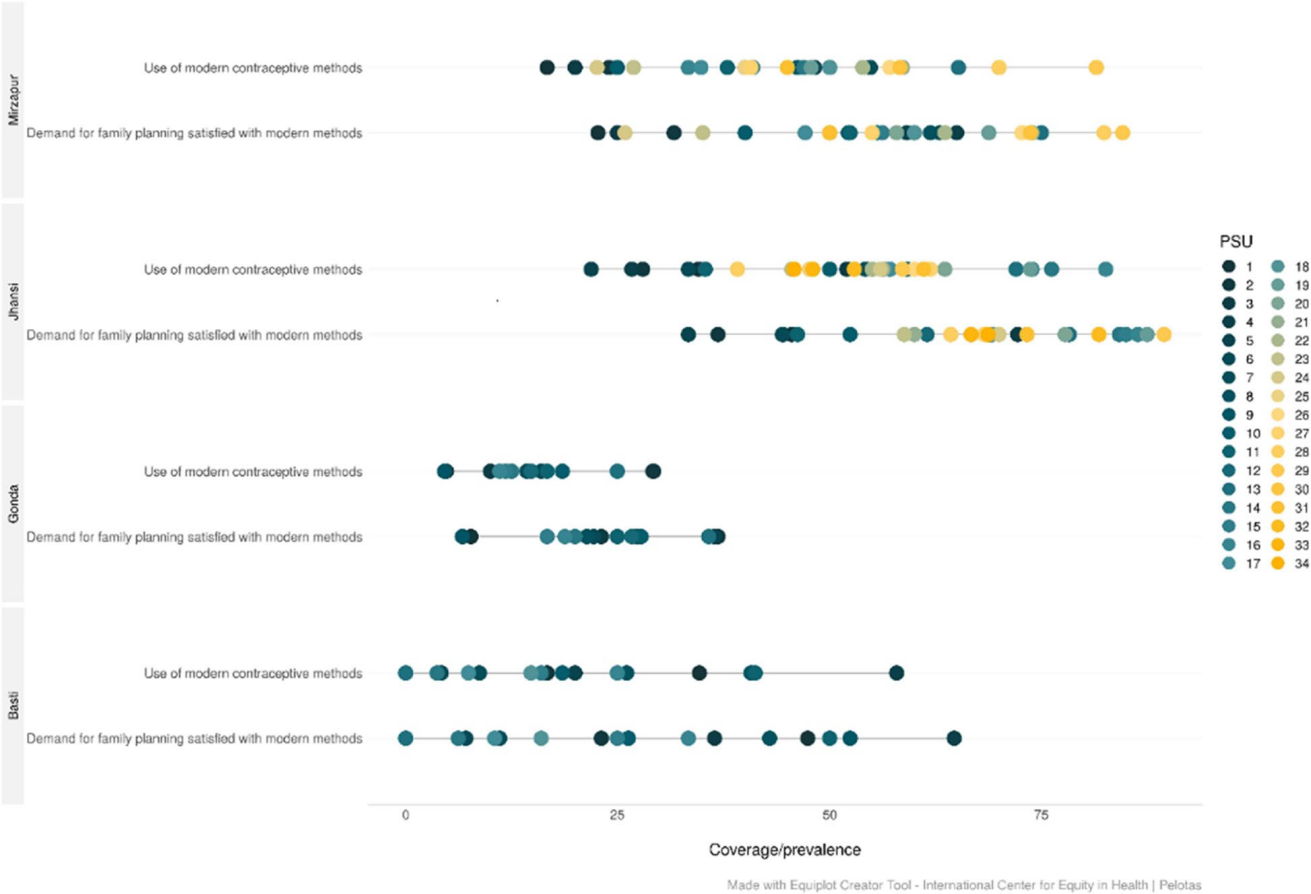
Distribution of within division inequalities in demand satisfied by modern contraception in selected divisions in Uttar Pradesh, India, 2021

Figure 3 indicates within division inequalities by ASHA areas in current use of modern contraceptive methods and demand met by modern contraception. Both the coverage indicators show significant inequalities by ASHA areas in all most all divisions except for Gonda division where both coverage and inequalities are low. However, the demand met by modern contraception in Gonda division shows clusters of ASHA areas in both the low and high family planning coverage. To better understand the range of within division inequalities in coverage of modern contraception, based on figure 3, two divisions with high modern contraceptive coverage and low inequality (Jhansi and Mirzapur), Gonda with low coverage and low inequity, and Basti with low coverage with high inequality are considered in figure 4. In figure 2, it is seen that Jhansi division experienced highest modern contraceptive coverage with lowest inequality compared to other divisions; however, figure 4 shows that the range of coverage within the division by ASHA areas is 25% to 75%. In fact, for some ASHA areas in Jhansi division the family planning demand satisfied for modern contraception ranged from more than 85% to less than 22% (Figure 4). Similarly, the Gonda division with lowest coverage and lowest inequity for demand satisfied for modern contraception (Figure 4) has some ASHA areas with less than 5% and more than 36% coverage (Fig. 4).

**Fig. 5:**
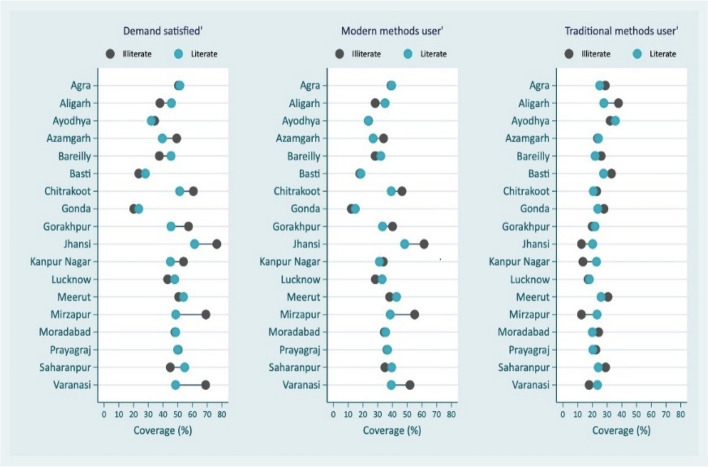
Divisional inequalities in family planning methods by education in Uttar Pradesh, India, 2021

Figure 5 presents the family planning coverage and education related inequalities in coverage indicators by administrative divisions of UP with different patterns. The first sub-figure reveals that demand satisfied by modern contraception among illiterates tend to be higher than literates. For some divisions such as Mirzapur, Jhansi and Varanasi, both the coverage and inequality by education levels are higher but the pattern is the same. For instance, in case of Mirzapur and Varanasi, the demand satisfied among the illiterates is 69% and the corresponding percentage for literates is 49%. Similar inequality patterns can be observed among modern contraceptive users by their education with higher coverage in Jhansi, Mirzapur and Varanasi divisions. However, among traditional contraceptive methods users by their education seem to indicate that there is no clear pattern. It should be also mentioned that there are some missing data in the figure due to smaller sample sizes.

**Fig. 6:**
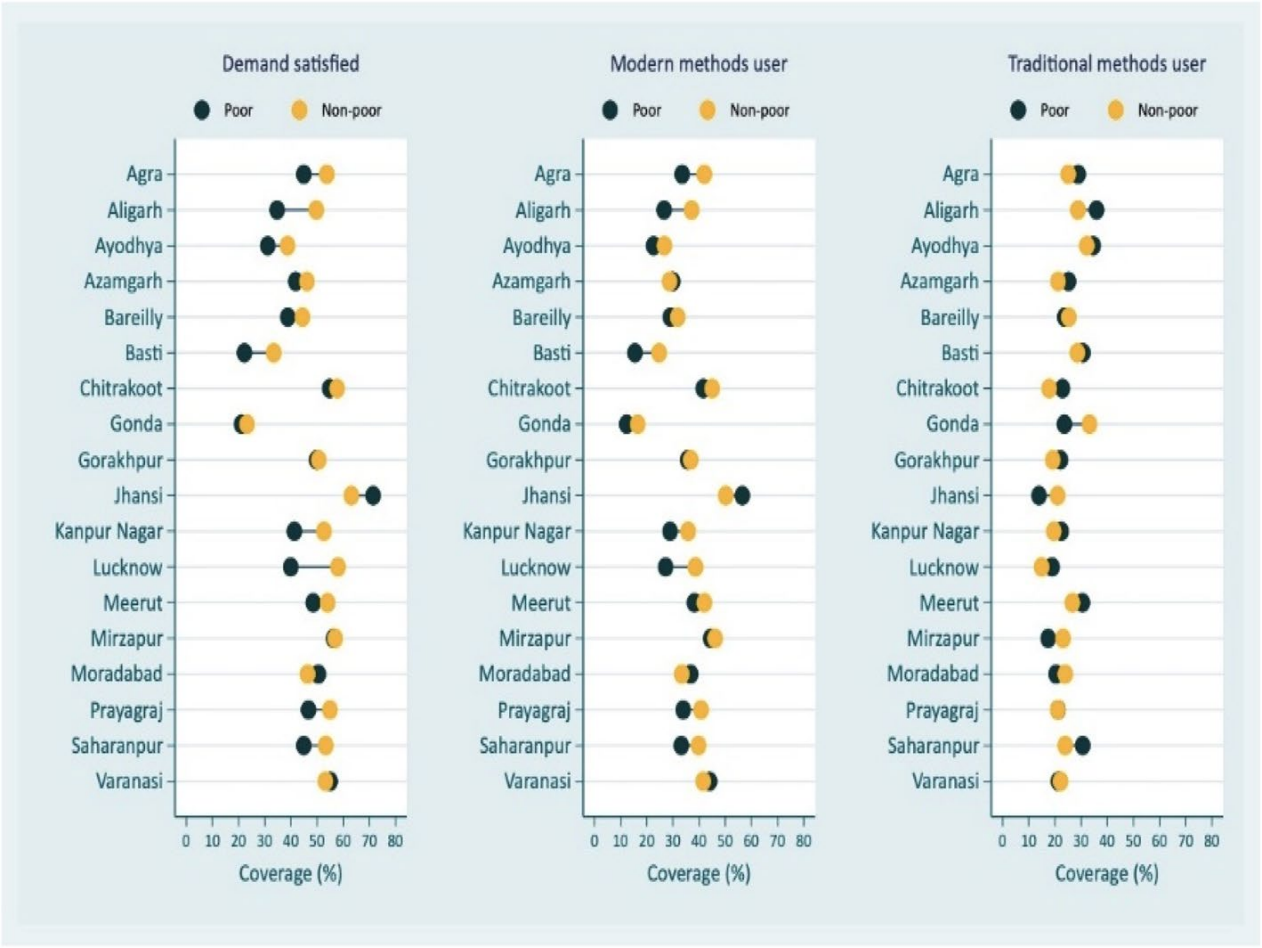
Divisional inequalities in family planning methods by income in Uttar Pradesh, India, 2021

Figure 6 presents the inequality in coverage of contraception by income level among administrative divisions in UP. Except Jhansi division, in almost all divisions, modern contraceptive usage is higher among rich couples in reproductive period. It is also observed that there are inequalities within a few divisions in usage of modern contraception between poor and rich in UP. For instance, in case of Lucknow division, demand satisfied for modern contraceptive methods is 39.9% among poor compared to 58.0% in case of rich. Even in case of Jhansi division where the coverage for modern family planning methods is the highest, inequality persists. The divisional pattern of inequalities is the same irrespective of the family planning coverage indicator for modern contraception in UP. As far as traditional family planning methods are concerned, within divisional differences between poor and non-poor are small though there is a tendency that more poor people use these methods with some exceptions. One of the exceptions is Gonda division where 23.6% of poor use the traditional methods compared to 33.2% non-poor.

**Fig 7:**
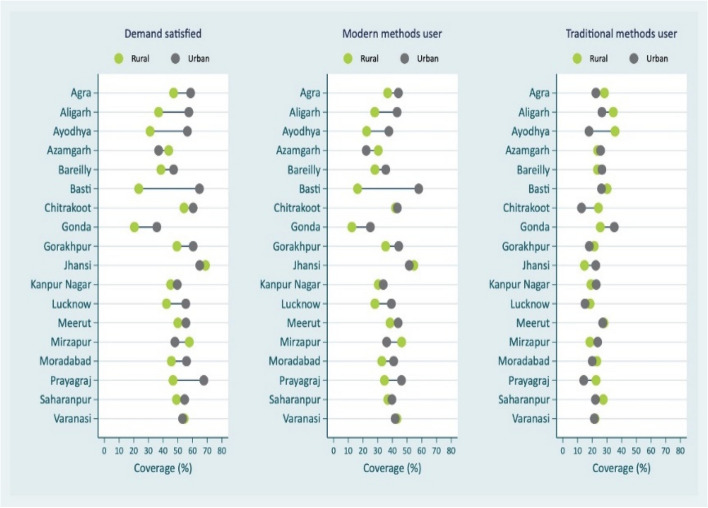
Divisional inequalities in family planning methods by place of residence in Uttar Pradesh, India, 2021

Figure 7 presents the inequity in coverage of contraception by place of residence among administrative divisions in UP. Except 2-3 divisions, in all other divisions the modern contraceptive usage is higher among urban residents. It is also observed that there are inequalities within divisions in usage of modern contraception between rural and urban residents in UP. For instance, in Basti division where the coverage for modern contraception is lower, demand satisfied for modern contraceptive methods is 16.3% among rural residents compared to 57.9% in case of urban residents. In Jhansi division where the coverage for modern family planning methods is the highest, place of residence does not seem to matter in accessing the modern methods. As far as traditional family planning methods are concerned, within division differences between rural and urban residents are small and difficult to establish a clear pattern. However, in case of Ayodhya division, it is observed that 35.5% of the rural residents use the traditional methods compared to 17.8% of urban residents.

## Discussion

Using data from the recent Family Planning Survey conducted in UP, the present study used different family planning coverage indicators with a special focus on demand satisfied by modern methods to identify the coverage gaps between and within administrative divisions. Between and within the divisions the inequalities were observed in family planning coverage irrespective of different coverage indicators. The inequity in family planning demand satisfied by divisions into four quadrants was useful in identifying Jhansi division as the highest family planning demand satisfied with lowest inequality; Basti as the division with the lowest family planning coverage with one of the highest inequalities and in Gonda division both demand satisfied for modern family planning methods and corresponding inequalities are one of the lowest. Figs. 5, 6 and 7 revealed intersectionality of education, wealth, place of residence and geographic divisions in identifying inequality patterns. There are no noticeable divisional differences in demand satisfied by modern contraceptive methods between literate and illiterate women, except in the case of Jhansi, Mirzapur and Varanasi divisions for illiterate women, the coverage is better (Fig. 5 ). These divisions indicate reverse pattern in case of traditional family planning methods use among women by education. However, further analysis of the data revealed changes that have occurred in use of sterilization and condom across divisions in UP from 2015-16 to 2021. According to the results, there are 7 divisions in UP that experienced decline in sterilization use and increase in condom use while 8 divisions experienced increase in both sterilization and condom use. The notable divisions are Jhansi with a 6% decline in sterilization use while 7% increase in condom use; on the other hand, Prayagraj division experienced 6% increase in condom use and Varanasi division with 8% increase in sterilization use. Additionally, stock-out of commodities is the important factor in the poor use of modern methods. Stock-out and limited contraception method availability limit method options, forcing people to choose methods that may or may not meet their preferences and needs. The results showed that higher modern methods use geographies have lesser stock-out of condom/oral contraceptive pills among frontline health care workers than those who belonged to poorer modern method use areas. It is also observed that there is increasing demand for other modern spacing methods such as Injectable/Antara including in poor performing divisions such as Gonda and Basti (results are not presented in the paper but available on request). It is probably due to lesser stock-out of commodities with ASHAs in their geographies, modern contraceptive methods such as condom use was higher. As expected, in most of the divisions, poor and rural residents are in a disadvantaged position compared to their respective counterparts in family planning demand satisfied for modern methods (Figs 6 and 7).

The success story of family planning demand satisfied by modern methods in Jhansi division is intriguing. Unlike Western Uttar Pradesh, Jhansi division or Bundelkhand region is not economically developed and, in many respects as bad as Eastern Uttar Pradesh [20, 21]. In fact, one of the lowest demand satisfied divisions, Aligarh, is part of economically developed region, Western Uttar Pradesh [20, 21]. Researchers have also shown that Jhansi division or Bundelkhand region has the lowest fertility rate in Uttar Pradesh [22]. It is difficult, if not impossible, to explain this anomaly in the absence of recent research. According to UP TSU FP survey, female sterilization and condom are the most preferred modern methods of contraception in UP and it is more so in Jhansi division irrespective of education and residence [23]. In our field visits (some authors were involved in FP Survey data collection as well as maternal and child health programming), it is observed that the demand is met by highly motivated service providers in public health care facilities. The motivation is there among both the young and senior doctors, and it seems, exemplary leadership of the senior providers seems to be having positive influence on the young doctors. The younger doctors tend to be locals and derive lots of pride and satisfaction in serving their community members. There seems to be a healthy competition in performing a maximum number of sterilizations, and it is re-reinforced by recognizing the best-performing service providers and front-line workers in an annual district level function attended by all healthcare employees. The front-line workers such as ASHAs and Auxiliary Nurse Midwives (ANMs) are making sure that community members can access the services. However, as indicated earlier, in recent years, in Jhansi division demand for sterilization has declined but demand for condoms has increased.

The status of women also might have played a role in effective use of modern contraception in reducing fertility to a replacement level in Jhansi division. For instance, unlike in other parts of UP, in Jhansi division, women participate in agricultural activities. Women in this division actively participate and, in many cases, lead agricultural cultivation. There is also historical evidence to show that women in this region are better empowered. According to Jhansi history, Jhansi was ruled by the Queen, Rani Lakshmi Bai, after her husband died [24]. Indian history books indicated that soon after her marriage, Rani Lakshmi Bai put together the women wing of Army as she believed protection of Jhansi Kingdom is not just the responsibility of men. Consequently, her women army wing took an active role in fighting the British invaders towards end of 19^th^ century. Moreover, Jhansi division or Bundelkhand region is different from rest of UP and more like border districts of Madhya Pradesh state and hence, there was a bill introduced in the parliament to form Bundelkhand region as a separate state by including border districts of Madhya Pradesh [20]. It is not surprising that the sterilization uptake in Jhansi division is about 50% and same as in Madya Pradesh. Whereas in case of India it is about 35% but in case of UP it is about less than 18% [22]. Another contributing factor in this region could be, the smaller proportion of Muslim population, around 5% compared to more than 17% in UP. Generally, amongst Muslims, the usage of modern contraception is lower [25], however, Muslim couples in this region seem to have influenced by the regional sub-cultural norms and are better motivated to practice modern contraception.

The worst performing divisions in terms of family planning demand satisfied are Gonda and Basti (Fig. 2) and these are part of Tarai belt, border to Nepal. Divisions in Tarai region recorded lower mCPR and predominantly traditional family planning methods users [23]. These are backward divisions, and modern family planning services, especially female sterilization, are not easily available [26, 27]. Since Public sector is the predominant service provider of modern methods including sterilization and condom in UP , accessibility of these services is also a problem because of out-of-pocket expenditure on public transportation to visit the facilities [19, 23]. Moreover, the couples of these divisions cannot afford to buy condoms from private facilities either. As seen in case of Basti, the modern contraception demand met is only 16% in rural areas. Since these divisions are predominantly rural and hence, it is not surprising that mCPR is much lower. In addition to the data presented above, recent research indicates that poorer people are more likely to use traditional family planning methods and in more developed Western region, couples are 19% less likely to use the traditional methods [8, 23]. Due to lack of accessibility to modern contraceptive methods, it is not surprising that couples from these divisions predominantly use traditional methods [23]. Recent research conducted in UP suggests that supply issues play an important role for increasing use of traditional methods [23, 27]. Since, the family planning services in UP are mostly available in Public Health Facilities, only 25% of the facilities provide female sterilization services, 11% male sterilization and 83% provide condoms [23, 28]. Hence, it is not surprising that in Basti and Gonda divisions, condoms and traditional methods are more being used [23].

To demonstrate the persistence of inequity both in best and worst performing divisions, figure 4 is useful. The best performing division, Jhansi, the demand met for modern family planning methods by ASHA areas (very small areas with population of about 1000) varies from as high as 85% to less than 22%. Similarly, the Gonda division with lowest coverage and lowest inequity for demand satisfied for modern contraception, there are some ASHA areas with less than 5% and more than 36% coverage. The situation is no different for other two divisions presented in figure 4, Mirzapur and Basti. In fact, there are quite a few ASHA areas in Basti division are doing as good as many ASHA areas in Mirzapur and Jhansi divisions but there is also an ASHA area where the coverage is almost nil (3.7%). India is a signatory to the 25th of September 2015 declaration of the United Nations General Assembly to adopt a resolution for the 2030 agenda for sustainable development goals, including goal 3, to ensure healthy lives and promote well-being for all at all ages [29, 30]. If the 2030 Countdown slogan “No One Left Behind” is to be achieved, the Governments of India and Uttar Pradesh cannot afford to focus only on worst performing divisions, Gonda and Basti, but also many ASHA areas of best performing divisions such as Jhansi and Mirzapur need attention as some of the ASHA areas are worse than Gonda and Basti divisions.

The divisions are not homogeneous in their socio-economic development [31], to throw some light on socio-economic inequalities, based on the recent literature, important stratification variables considered are education, income, and place of residence [9, 19]. In case of Jhansi, Mirzapur and Varanasi divisions, the demand satisfied is higher among illiterates (Figure 5). This is consistent with the previous research. [9, 13] Indian Family Planning Program has been successful in its efforts to popularize a small family norm, and couples are motivated to access contraceptive services to achieve their desired family size. This does not mean that economic resources and being in urban areas are not relevant in accessing and utilizing modern contraceptive methods. In one of the worst coverage divisions such as Basti, the demand met by modern contraception in urban areas is 60% compared to only 16% in rural areas. These above results are also corroborated by district level analysis of fertility decline by others [22]. The study has shown that the sterilization usage has increased substantially in very low and middle TFR clusters, while the contribution of the same method has declined, and traditional method has increased in high TFR cluster [22]. According to the study, it is due to lack of availability and accessibility of preferred method of choice. It also seen in case of High Priority Districts (these are disadvantaged districts defined by Government of India) and non-High Priority Districts comparison, one of the factors contributing to the decline in fertility in very low TFR and middle TFR clusters is increase in the usage of sterilization [22, 32]. The patterns in figure 6 reveal that the poor couples are less likely to use modern methods, and this is indicative of lack of affordability. Even if the modern contraceptives are provided free in government facilities, the facilities are not easily accessible to rural and poor people as the services tend to be in higher facilities away from their place of residence. The out-of-pocket expenditure on public transportation to reach the facilities is an issue for these couples. Therefore, family planning programmes in UP need to continue with emphasis on improving availability, accessibility, utilization, quality of care, counselling, and better management of side effects of all modern methods, specially to meet the target by 2030 of “No One Left Behind”.

## Conclusions

The main finding of the present study is that the inequality exists in modern family planning methods’ coverage in Uttar Pradesh even in best and worst performing divisions and even in extremely smaller geographies such as ASHA areas. Within geographies, there are also inequalities by education, wealth, and place of residence. These are important factors to consider for policymakers for effective resource allocation and utilization. If the government of India must achieve the goal of “No One Left Behind” by 2030, the main reasons affecting adequate coverage of modern family planning methods such as availability, accessibility, acceptability, and utilization of modern family planning methods by disadvantaged geographies and groups must be addressed. The number of facilities that can offer high quality postpartum and birth interval contraceptive services must be increased, especially Community Health Centres and Primary Health Centres as these facilities will make services more accessible to rural populations. Currently, there are more than 30,000 public health facilities; these are not enough compared to the prescribed norms (national norms based on rural population as defined by Indian Public Health Standards). The shortfall across different types of facilities i.e. Sub Centers, Community Health Centres and Primary Health Centres range between 42% to 51% as per Rural Health Statistics [28]. Concomitant with this, the severe shortage of of critical clinical and non-clinical resources, e.g., about 25% of the sanctioned Health Worker (female) positions at SCs and PHCs in rural areas are vacant as per Rural Health Statistics [28]. The traditional approaches to programming may have to be re-examined. For instance, applying advanced epidemiological methodologies to more robust hotspot identification and combinations of scientifically proven, cost-effective, and scalable interventions targeted to identified populations in geographic areas may greatly improve the adequate coverage of the modern methods in UP. It may also be necessary to sensitize front–line workers and other health care providers to address any barriers against socio-economically disadvantaged communities’ in accessing and of using modern contraceptive methods. Overall, direct information to women and couples using digital tools and technology needs to be explored to improve access to comprehensive and accurate information, tailored recommendations and for follow-up and reminders .

## Data Availability

NFHS-5 data set is publicly available and UP TSU FP Survey data is available on request.

## Appendix

**Table:** Demand satisfied by modern methods (IFPS, 2021)

Division	Modern method users	Traditional method users	Unmet need	Non-Users and no unmet need	Demand satisfied by modern methods	N
Agra	39.4	26.2	11.5	23.0	51.1	811
Aligarh	31.9	32.3	11.4	24.3	42.2	772
Ayodhya	23.7	34.1	14.1	28.2	32.9	533
Azamgarh	29.5	23.9	15.0	31.6	43.1	686
Bareilly	29.9	24.3	19.4	26.5	40.6	603
Basti	18.2	30.0	22.4	29.4	25.8	438
Chitrakoot	42.4	21.7	12.4	23.6	55.5	799
Gonda	13.2	26.1	22.0	38.7	21.6	376
Gorakhpur	36.1	21.0	14.7	28.1	50.3	694
Jhansi	53.6	17.2	8.6	20.7	67.5	788
Kanpur Nagar	31.8	20.6	15.3	32.3	46.9	562
Lucknow	31.0	17.6	19.0	32.4	45.8	551
Meerut	41.2	27.5	9.3	22.0	52.8	801
Mirzapur	44.9	19.1	15.4	20.5	56.5	791
Moradabad	35.0	22.2	15.1	27.7	48.4	716
Prayag Raj	36.6	21.1	15.4	26.9	50.0	657
Saharanpur	37.7	26.0	10.7	25.6	50.7	802
Varanasi	42.9	21.8	14.5	20.8	54.1	820
Total	33.9	23.7	15.1	27.4	46.7	12,200

## Abbreviations

ANM: Auxiliary Nurse Midwives
ASHA: Accredited Social Health Activist
CEB: Census Enumeration Blocks
CMW: Currently Married Women
FLW: Front Line Workers
FP: Family Planning
HC-HI: High Coverage: High Inequity
HC-LI: High Coverage- Low Inequity
LC-HI: Low Coverage: High Inequity
LC-LI: Low Coverage -Low Inequality
IHAT: India Health Action Trust
IIPS: International Institute for Population Sciences
mCPR: Modern Contraceptive Prevalence Rate
CPR: Contraceptive Prevalence Rate
NFHS: National Family Health Surveys
NOLB: No One Left Behind
NRHM: National Rural Health Mission
OBC: Other Backward Castes
PPS: Probability Proportional to Size
PSU: Primary Sampling Units
SC: Scheduled Caste
SCs: Sub Centers
STATA: Statistical Software
PHC: Primary Health Center
SDG: Sustainable Development Goals
SIFPSA: State Innovations in Family Planning Services Project Agency
ST: Scheduled Tribe
TFR: Total Fertility Rate
TM: Traditional Methods
UoM: University of Manitoba
UP: Uttar Pradesh
UP TSU: Uttar Pradesh Technical Support Unit
